# Pars Plana Vitrectomy and ILM Peeling for Refractory Diabetic Macular Edema Without Vitreomacular Traction

**DOI:** 10.3390/jcm14113686

**Published:** 2025-05-24

**Authors:** Sylvain el-Khoury, Chloe Ngo, Marc Muraine, Youssef Abdelmassih, Alexandre Portmann

**Affiliations:** 1 SOS Rétine Sud, 29 Boulevard de la Ferrage, 06400 Cannes, France; 2 Department of Ophthalmology, University Hospital Rouen, 37 Boulevard Gambetta, 76000 Rouen, France; 3 Department of Retina and Pediatric Ophthalmology, Rothschild Foundation Hospital, 29 rue Manin, 75019 Paris, France

**Keywords:** intravitreal injection, anti-VEGF, dexamethasone implant, diabetic macular edema, ILM peeling, pars plana vitrectomy

## Abstract

**Background**: The aim of this study was to evaluate the outcome of pars plana vitrectomy (PPV) with internal limiting membrane (ILM) peeling in patients with diabetic macular edema (DME) refractory to intravitreal injections (IVIs) and without vitreomacular traction. **Methods:** In this retrospective consecutive case series, we included patients that underwent PPV with ILM peeling for refractory DME. Examination was performed at baseline, and at the 1- and 6-month follow-up. Primary endpoints were best-corrected distance visual acuity (BCVA) and central macular thickness (CMT). **Results:** A total of 15 eyes were included, and BCVA improved from 0.69 ± 0.27 logMAR preoperatively to 0.48 ± 0.28 logMAR at the 1-month (*p* = 0.013) follow-up and 0.42 ± 0.29 logMAR at the 6-month (*p* < 0.001) follow-up. At the 6-month follow-up, 10 eyes (66.6%) gained at least two lines of vision. The BCVA of pseudophakic eyes (nine eyes) improved from 0.64 ± 0.21 logMAR at baseline to 0.40 ± 0.26 logMAR at the 6-month follow-up (*p* = 0.02). CMT decreased from 457 ± 114 µm preoperatively to 336 ± 112 µm at the 1-month (*p* = 0.035) follow-up and 302 ± 68 µm at the 6-month (*p* = 0.001) follow-up. During follow-up, only two eyes received IVIs: one following vitreous hemorrhage and one for persistent DME. **Conclusion:** PPV with ILM peeling improves BCVA and reduces CMT in eyes with DME refractory to IVIs up to the 6-month follow-up.

## 1. Introduction

Diabetic retinopathy remains a leading cause of blindness worldwide, particularly among working-age adults, with diabetic macular edema (DME) being a major contributor to legal blindness in patients with type 2 diabetes [1]. The prevalence of DME varies depending on the study and population, with a recent U.S. study reporting a prevalence of 3.8% [2]. DME results from the breakdown of the blood–retinal barrier, leading to an imbalance between retinal fluid exudation and resorption. Key risk factors include chronic hyperglycemia and retinal ischemia, which induce oxidative stress, inflammation, hypoxia, and vascular dysfunction. These pathological processes stimulate the release of vascular endothelial growth factor (VEGF) and other cytokines, which disrupt the inner blood–retinal barrier and cause fluid accumulation in the outer plexiform and inner nuclear layers [3].

Several studies have highlighted the potential role of the vitreous, posterior hyaloid, and internal limiting membrane (ILM) in the pathogenesis of DME. Both tangential and anteroposterior vitreomacular traction (VMT) have been associated with DME, suggesting a potential mechanical component [4]. Moreover, spontaneous resolution of DME has been observed following complete posterior vitreous detachment (PVD) [5]. ILM peeling may help reduce mechanical stress at the vitreoretinal interface, relieve tangential traction, improve retinal oxygenation, enhance microcirculation, and facilitate the clearance of intra-retinal cytokines and VEGF [6,7]. A small study has supported this theory, showing improvement in refractory DME after ILM removal in eyes previously treated with vitrectomy without ILM peeling [8].

Recent research has also shown that vitreoretinal interface abnormalities, such as epiretinal membranes (ERMs) and VMT, may diminish the efficacy of anti-VEGF therapy in DME [9]. Numerous studies have evaluated the role of pars plana vitrectomy (PPV) in DME, with growing evidence suggesting that PPV is effective in selected cases—particularly in eyes with VMT [10,11]. However, most existing studies focus on patients with VMT and were conducted prior to the widespread use of smaller gauge vitrectomy techniques and intravitreal injections (IVIs) of anti-VEGF agents.

The aim of the present study is to evaluate the efficacy of 23-gauge PPV combined with ILM peeling in patients with DME without signs of VMT or ERM and who are refractory to IVIs.

## 2. Materials and Methods

In this is monocentric, retrospective study we included patients that underwent PPV with ILM peeling for DME refractory to IVIs of anti-VEGF and/or dexamethasone at the Rouen University Hospital, France, between September 2015 and July 2017. Inclusion criteria were as follows: patients with type 2 diabetes, a central macular edema with a central macular thickness (CMT) >300 μm as measured by spectral-domain optical coherence tomography (SD-OCT), absence of ERM or VMT, and no history of focal macular laser treatment. All included patients had undergone complete pan-retinal photocoagulation (PRP) for severe non-proliferative or proliferative diabetic retinopathy prior to surgery. Written informed consent was obtained from all participants, and the study adhered strictly to the tenets of the Declaration of Helsinki. The research was approved by the Rouen University Hospital review board under the study number E-2025-03.

Refractory DME was defined as the persistence of a macular edema one month after receiving at least three monthly IVIs of anti-VEGF or two months after the injection of at least one dexamethasone implant (Ozurdex^®^, Allergan Inc., Irvine, CA, USA).

At baseline and at the 1-month and 6-month postoperative follow-ups, all patients underwent a comprehensive ophthalmological evaluation, which included best-corrected distance visual acuity (BCVA), funduscopic examination, and SD-OCT imaging (Cirrus HD-OCT, Carl Zeiss Meditec, Dublin, CA, USA). The primary outcome measures were BCVA and CMT assessed 6 months postoperatively. BCVA was recorded using the Snellen chart and converted to logMAR scale for statistical analysis. Any additional intravitreal injections administered after surgery and any postoperative complications were also recorded.

All surgical procedures were performed under peribulbar or general anesthesia by a single experienced surgeon (AP). A 23-gauge, 2-port PPV was conducted using the DORC EVA system (DORC, Zuidland, The Netherlands). A PVD was induced; ILM was stained using dual blue dye (DORC, Zuidland, The Netherlands), and peeled using the Alcon Grieshaber end-grasping asymmetrical forceps (Alcon, Fort Worth, TX, USA) over an area of approximately three papillary diameters surrounding the fovea. At the conclusion of surgery, fluid–air exchange was performed, and patients were left under air. The sclerotomies were left unsutured. For phakic patients, a combined surgical approach involving PPV and phacoemulsification was undertaken.

### Statistical Analysis

The SPSS program version 24.0 (IBM Corporation, Armonk, NY, USA) was used for statistical analysis. Descriptive statistics were reported as percentage for categorical variables, as mean ± standard deviation (SD) (median; range) for continuous variables. The Wilcoxon test was used to compare pre- and postoperative BCVA and CMT. The association between preoperative and 6-month postoperative BCVA was evaluated using Pearson correlation coefficient. A *p*-value < 0.05 was considered statistically significant.

## 3. Results

A total of 15 eyes of 15 patients were included in this study. The mean age at the time of surgery was 65.5 ± 8.5 years (median: 65.0; range: 50–79 years). Nine eyes were pseudophakic at the time of surgery, while the remaining six underwent combined cataract extraction and PPV. The mean duration of DME prior to surgery was 10.9 ± 8.4 months. Baseline characteristics of the included patients are summarized in Table 1.

Overall, BCVA significantly improved from 20/100 (0.69 ± 0.27 logMAR; median: 0.70; range: 0.30–1.30) at baseline to 20/60 (0.48 ± 0.28 logMAR; median: 0.50; range: 0.10–1.00; *p* = 0.013) at the 1-month follow-up and to 20/53 (0.42 ± 0.29 logMAR; median: 0.40; range: 0.00–1.00; *p* < 0.001 compared to baseline, *p* = 0.33 compared to 1 month) at the 6-month follow-up. Ten eyes (66.6%) achieved at least a two-line BCVA improvement at the 6-month postoperative follow-up. Eyes with normal outer retina on OCT had significantly better BCVA at both the 1-month (0.15 ± 0.1 logMAR vs. 0.60 ± 0.21 logMAR; *p* = 0.001) and 6-month follow-up (0.13 ± 0.19 vs. 0.53 ± 0.25 logMAR; *p* = 0.01). Table 2 reports the effect of outer retina status on baseline characteristics and outcomes.

When considering only the pseudophakic subgroup at the time of surgery, BCVA also showed improvement. The mean BCVA in this subgroup was 20/87 (0.64 ± 0.21 logMAR; median: 0.70; range: 0.40–1.00) at baseline, 20/60 (0.48 ± 0.29 logMAR; median: 0.50; range: 0.10–1.00; *p* = 0.15) at the 1-month follow-up, and 20/50 (0.40 ± 0.26 logMAR; median: 0.40; range: 0.00–0.80; *p* = 0.02 compared to baseline, *p* = 0.43 compared to 1 month) at the 6-month follow-up.

**Figure 1:**
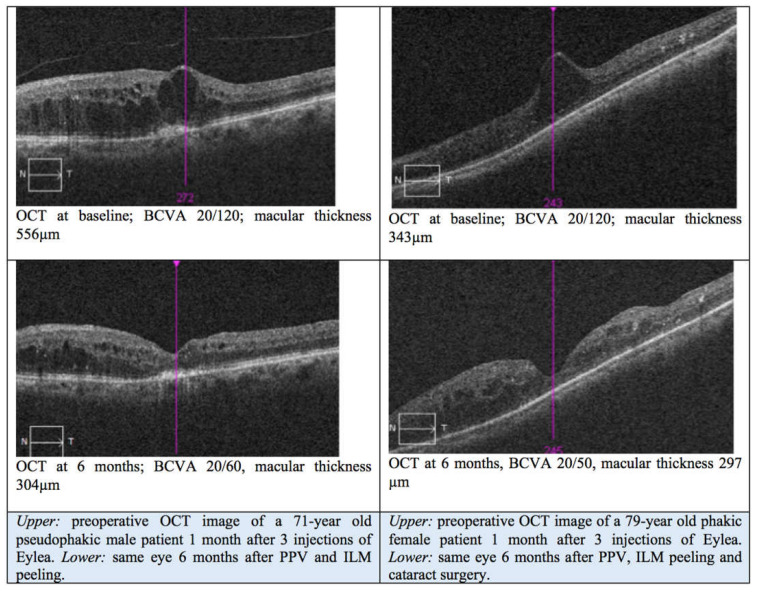
OCT images of two eyes with diabetic macular edema (DME) at baseline (superior images) and at the 6-month postoperative follow-up (inferior images). BCVA: best-corrected visual acuity; ILM: internal limiting membrane; PPV: pars plana vitrectomy.

CMT also decreased significantly after surgery. The mean CMT was 457 ± 114 µm (median: 442; range: 305–816) at baseline, 336 ± 112 µm (median: 310; range: 177–575; *p* = 0.035) at the 1-month follow-up, and 302 ± 68 µm (median: 304; range: 200–482; *p* = 0.001 compared to baseline, *p* = 0.089 compared to 1 month) at the 6-month follow-up. A summary of the results is provided in Table 3, and Figure 1 illustrates two exemplary cases both preoperatively and at the 6-month postoperative follow-up.

A positive correlation was observed between preoperative BCVA and BCVA at the 6-month follow-up (Pearson correlation coefficient r = 0.82, *p* < 0.001), suggesting that better baseline visual acuity was associated with improved surgical outcomes. However, no significant correlation was found between CMT and BCVA at any timepoint (preoperative or 1-month or 6-month follow-up). A positive correlation was found between the duration of DME and BCVA at baseline and the 1-month and 6-month follow-up (*p* = 0.001, 0.048, and 0.002, respectively). Table 4 reports the correlation between baseline characteristics and outcomes.

No intraoperative complications were reported. During the follow-up period, one eye developed an intravitreal hemorrhage that required surgical removal with an IVI of avastin. At the six-month follow-up, one eye with persistent DME received an IVI of dexamethasone, despite showing a modest improvement in BCVA of 0.1 logMAR and a reduction of 70 µm in CMT.

## 4. Discussion

The present study demonstrated that ILM peeling combined with PPV for refractory DME resulted in improved BCVA, reduced CMT, and decreased recurrence of macular edema over a follow-up of six months. On average, BCVA improved by 0.27 logMAR across all eyes, equivalent to a gain of 14 ETDRS letters, and by 0.24 logMAR in pseudophakic eyes, corresponding to 12 ETDRS letters. Ten eyes (66.6%) gained at least two lines following surgery. The outcomes of PPV with ILM peeling are controversial in the literature. Some studies report significant BCVA improvements after surgery, with 43% to 92% of eyes gaining at least two lines, while others, although noting a decrease in CMT, did not observe significant visual improvement [12,13,14,15]. Yanyali et al. reported improvements in both retinal thickness and visual acuity after ILM peeling [16]. In contrast, Hoerauf et al. observed a reduction in DME without a corresponding gain in visual acuity in their prospective trial [17]. This discrepancy may be attributed to several factors, including progression of cataract during follow-up, duration of macular edema prior to surgery, variability in macular ischemia, prior treatment history (IVI or laser photocoagulation), and severity of diabetic retinopathy.

In the present study, we found a positive correlation between preoperative BCVA and BCVA at the 6-month follow-up. However, no correlation was found between CMT and BCVA. The lack of correlation could be due to the fact that both very low and very high CMT values are associated with poor visual acuity, as they represent either retinal atrophy or persistent edema. Kunikata et al. reported a significant correlation between preoperative and postoperative BCVA at 6 months following idiopathic ERM removal (Spearman’s r = 0.44, *p* < 0.001) [18]. Guo et al. found a moderate positive correlation between BCVA following ILM peeling for DME and both preoperative CMT and postoperative macular edema degree [19]. Several studies have shown that postoperative BCVA was not correlated with postoperative CMT [20,21]. Although the 6-month follow-up demonstrated significant improvements in anatomical and functional outcomes, this duration may not be sufficient to evaluate long-term results or recurrence rates. Gunay and Erdogan showed that although PPV with ILM peeling was effective in DME, the efficacy tended to decrease with time, with up to 55% of eyes needing additional treatment beyond the 2-year follow-up [22].

Anti-VEGF IVI remains the gold standard treatment for DME except in the presence of VMT or ERM [23]. Although the visual gain is good with IVI, most patients need to follow a strict treatment protocol with multiple visits decreasing the patient’s compliance and resulting in lower visual recovery. Furthermore, the access to IVIs could be limited for patients in certain settings or due to financial constraints [24]. Additionally, around 40% of patients may suffer from persistent DME requiring monthly IVIs [25,26]. Several mechanisms have been proposed to explain the role of PPV in DME treatment. These include the relief of tractional forces caused by vitreomacular adhesion, VMT, or ERM, improved retinal oxygenation and increased perifoveal capillary blood flow, and a reduction in inflammatory and angiogenic mediators such as VEGF, histamine, and free radicals in the pre-retinal space after posterior hyaloid removal [4,27,28,29,30].

Adding ILM peeling improves the efficacy of the PPV in eyes with DME. A metanalysis showed that PPV with ILM peeling was associated with a greater improvement in BCVA (OR = 1.66; *p* = 0.01) and a higher rate of CMT reduction (OR = 3.89; *p* = 0.01) compared to PPV alone [15]. The ILM in diabetic patients is thicker than in non-diabetic individuals, due to increased expression of collagen, fibronectin, and laminin [31,32]. This increased thickness alters the dynamic exchange between the vitreous and retina [33]. Moreover, the pre-macular vitreous pocket adheres strongly to the ILM, and PVD alone may not be sufficient to eliminate this pocket, making ILM peeling necessary for complete hyaloid removal [8,34,35,36]. Additionally, the ILM acts as a scaffold for astrocyte proliferation. Its removal helps inhibit glial proliferation and reduces the risk of ERM formation [8]. Postoperative ERM formation following PPV without ILM peeling is reported in about 10% of cases, while it is almost nonexistent after ILM peeling [37]. These benefits were confirmed by Stefaniotou et al. in a retrospective study and later by Hoerauf et al. in a prospective randomized trial with an extended follow-up [17,36]. The injury to Müller cell footplates during ILM peeling initiates a repair process mediated by epidermal growth factor receptor, leading to vertical glial proliferation [38,39].

Initially proposed for refractory DME, PPV combined with ILM peeling has demonstrated anatomical benefits, though functional improvements in visual acuity have been variable [12,15,40,41]. Recently, this surgical approach has been explored in treatment-naïve DME patients, suggesting its potential as a primary intervention [42,43,44]. Patient selection plays a critical role in achieving favorable outcomes. Several studies have evaluated predictive factors for better postoperative outcomes. In our study, we found that patients with abnormal outer retina had a worse visual prognosis. Other studies have identified preoperative external limiting membrane integrity as a reliable predictor of visual improvement [45,46]. Ranno et al. found that the presence of subretinal fluid at baseline was associated with better visual recovery [14]. Additional positive prognostic indicators include better preoperative BCVA, lower baseline CMT, smaller foveal avascular zone diameter, and higher vessel density at the time of edema resolution [24,47,48]. Conversely, the presence of hyperreflective foci in the outer retinal layers has been associated with damage to the IS/OS junction and a worse visual prognosis [49,50,51,52,53].

There are several limitations to our study, including the small sample size, short follow-up duration, and absence of a control group receiving IVIs alone. It is noteworthy that most studies involving PPV for non-tractional DME have a limited number of included participants. In fact, in the VIDEO trial, the authors could only reach 42% of the enrollment goal over 32 months and could not find evidence to support a clinical benefit of PPV plus ILM peeling as an adjunct to a treat-and-extend regimen of anti-VEGF therapy for DME [54].

However, strengths include the availability of complete 6-month data for all patients and the fact that surgeries were performed by the same experienced surgeon. Although PPV with ILM peeling appears beneficial in selected cases of refractory DME without VMT or ERM, prospective randomized controlled trials directly comparing it to repeated IVIs are needed to validate these findings. The VVV-DME trial aims to evaluate early PPV as a primary treatment for treatment-naïve DME. Their protocol hypothesizes that early surgical intervention could lessen the cumulative injection burden, prevent diabetic retinopathy progression, and achieve more durable visual outcomes [26].

## 5. Conclusions

In conclusion, PPV with ILM peeling appears to be an effective treatment option for refractory DME without VMT, offering improved visual acuity and reduced macular thickness with a low complication rate. This approach may also help lower the risk of infection associated with repeated IVI and reduce overall treatment costs. However, to better evaluate its comparative efficacy versus repeated IVI, a prospective, randomized study with a larger patient cohort is necessary.

## Figures and Tables

**Table 1:** Baseline characteristics for all 15 eyes (15 patients). BCVA: best-corrected visual acuity; CMT: central macular thickness; IS/OS: inner segment/outer segment; SD: standard deviation; VEGF: vascular endothelial growth factor.

Baseline Characteristics	15 Patients (15 Eyes)
Gender	
Female (%)	7 (47%)
Male (%)	8 (53%)
Age in years	65.5 ± 8.5 (50–79)
Diabetes treatment	
Oral (%)	3 (20%)
Insulin (%)	12 (80%)
BCVA in logMAR [mean ± SD (range)]	0.69 ± 0.27 (0.30–1.30)
Intravitreal injections	
Anti-VEGF (%)	12 (80%)
Dexamethasone (%)	1 (7%)
Anti-VEGF + Dexamethasone (%)	2 (14%)
DME duration in months [mean ± SD (range)]	10.9 ± 8.4 (3.1–32.5)
Lens status	
Phakic (%)	6 (40%)
Pseudophakic (%)	9 (60%)
CMT in µm [mean ± SD (range)]	457 ± 114 (305–816)
Abnormal IS/OS junction	11 (73%)

**Table 2:** Baseline characteristics and outcomes according to outer retina status on OCT. BCVA: best-corrected visual acuity; CMT: central macular thickness; DME: diabetic macular edema; SD: standard deviation.

	Normal OCT	Abnormal OCT	*p*-Value
Age in years (mean ± SD)	67.3 ± 9.5	64.8 ± 8.5	0.66
Preoperative BCVA in logMAR (mean ± SD)	0.55 ± 0.17	0.75 ± 0.28	0.28
CMT in µm (mean ± SD)	396 ± 129	479 ± 146	0.41
Duration of DME in months (mean ± SD)	5.6 ± 3.5	12.9 ± 9.0	0.08
BCVA at 1 month in logMAR (mean ± SD)	0.15 ± 0.10	0.60 ± 0.21	0.001
CMT at 1 month in µm (mean ± SD)	334 ± 37	336 ± 133	0.45
BCVA at 6 months in logMAR (mean ± SD)	0.13 ± 0.19	0.53 ± 0.25	0.01
CMT at 6 months in µm (mean ± SD)	296 ± 28	304 ± 79	1
BCVA improvement at 6 months in logMAR (mean ± SD)	0.43 ± 0.05	0.22 ± 0.17	0.06
CMT improvement at 6 months in µm (mean ± SD)	101 ± 120	175 ± 152	0.66

**Table 3:** Evolution of CMT and BCVA at 1- and 6-month postoperative follow-up. BCVA is presented for all eyes and for pseudophakic eyes alone. BCVA: best-corrected visual acuity; CMT: central macular thickness; SD: standard deviation. * *p*-value refers to the comparison to the 1-month follow-up.

	Baseline	1-Month Follow-Up	6-Month Follow-Up
CMT in µm (mean ± SD)	457 ± 114	336 ± 112; *p* = 0.035	302 ± 68; *p* = 0.001, *p* * = 0.089
BCVA in logMAR (mean ± SD)	0.69 ± 0.27	0.48 ± 0.28; *p* = 0.013	0.42 ± 0.29; *p* < 0.001, *p* * = 0.33
Improvement ≥ 2 ETDRS lines (%)		10 (67)	10 (67)
Improvement 0–2 ETDRS lines (%)		0 (0)	4 (27)
No change (%)		2 (13)	1 (7)
Worsening 0–2 ETDRS lines (%)		2 (13)	0 (0)
Worsening ≥ 2 ETDRS lines (%)		1 (7)	0 (0)
BCVA in logMAR in pseudophakic eyes (*n* = 9) (mean ± SD)	0.64 ± 0.21	0.48 ± 0.29; *p* = 0.15	0.40 ± 0.26; *p* = 0.02, *p* * = 0.43
Improvement ≥ 2 ETDRS lines (%)		6 (67)	6 (67)
Improvement 0–2 ETDRS lines (%)		0 (0)	2 (22)
No change (%)		1 (11)	1 (11)
Worsening 0–2 ETDRS lines (%)		1 (11)	0
Worsening ≥ 2 ETDRS lines (%)		1 (11)	0

**Table 4:** Correlation between baseline characteristics and outcomes. BCVA: best-corrected visual acuity; CMT: central macular thickness; DME: diabetic macular edema.

	Duration of DME	Preoperative BCVA	Preoperative CMT
Preoperative BCVA [Pearson correlation coefficient (*p*-value)]	0.78 (0.001)	1	0.21 (0.46)
BCVA at 1 month [Pearson correlation coefficient (*p*-value)]	0.52 (0.048)	0.42 (0.12)	0.27 (0.32)
BCVA at 6 months [Pearson correlation coefficient (*p*-value)]	0.73 (0.002)	0.82 (<0.001)	0.41(0.13)
CMT at baseline [Pearson correlation coefficient (*p*-value)]	0.28 (0.32)	0.21 (0.46)	1
CMT at 1 month [Pearson correlation coefficient (*p*-value)]	0.19 (0.52)	0.03 (0.93)	0.01 (0.96)
CMT at 6 months [Pearson correlation coefficient (*p*-value)]	−0.01 (0.96)	−0.43 (0.11)	0.21 (0.46)
BCVA improvement at 6 months [Pearson correlation coefficient (*p*-value)]	−0.04 (0.90)	0.15 (0.59)	−0.37 (0.17)
CMT improvement at 6 months [Pearson correlation coefficient (*p*-value)]	0.28 (0.32)	0.41 (0.13)	0.89 (<0.001)

## Data Availability

The data are available from the corresponding author upon reasonable request.

## Abbreviations

The following abbreviations are used in this manuscript:

BCVA	best-corrected distance visual acuity
CMT	central macular thickness
DME	diabetic macular edema
ERM	epiretinal membranes
ILM	internal limiting membrane
IVI	intravitreal injection
PPV	pars plana vitrectomy
PVD	posterior vitreous detachment
SD	standard deviation
SD-OCT	spectral-domain optical coherence tomography
VMT	vitreomacular traction
